# Assessing the environment for engagement in health services: The Audit for Consumer Engagement (ACE) tool

**DOI:** 10.1111/hex.13610

**Published:** 2022-10-28

**Authors:** Ashfaq Chauhan, Bronwyn Newman, Ramesh Lahiru Walpola, Holly Seale, Elizabeth Manias, Carlene Wilson, Reema Harrison

**Affiliations:** ^1^ Centre for Health Systems and Safety Research, Australian Institute of Health Innovation, Faculty of Medicine, Health and Human Sciences Macquarie University North Ryde New South Wales Australia; ^2^ School of Population Health, Faculty of Medicine and Health, UNSW Sydney Kensington New South Wales Australia; ^3^ School of Nursing and Midwifery, Centre for Quality and Patient Safety Research, Institute for Health Transformation Deakin University Melbourne Victoria Australia; ^4^ Olivia Newton‐John Cancer Wellness and Research Centre Austin Health Heidelberg Victoria Australia; ^5^ School of Psychology and Public Health La Trobe University Bundoora Victoria Australia

**Keywords:** audit tool, consumer engagement, facility design, health service design, physical environment

## Abstract

**Introduction:**

Although it is widely accepted that the physical environment can impact health quality and care outcomes, its impact on consumer engagement with health services has not been examined. Currently, no tools exist that assess the opportunities for consumer engagement offered within the physical environment. We aimed to develop and validate an environmental audit tool to assist health services and researchers to assess the extent to which the physical environment in health services creates and supports opportunities for consumer engagement.

**Method:**

An iterative, team‐based approach in partnership with diverse stakeholders was used to develop the Audit for Consumer Engagement (ACE) tool. A four‐stage process consisting of desktop review, concept clarification, identification of domains and validation was implemented. The tool was validated in one cancer service via face and content validation and inter‐rater reliability was also assessed.

**Results:**

The ACE tool was demonstrated to comprise four main domains (access and signage information; resources for consumers; resources to support diversity; and consumer engagement events or activities) measured with 17 items. Face and content validity was achieved, and preliminary reliability analysis indicated substantial agreement between the two researchers for all four domains with an average of 86% agreement.

**Conclusion:**

The ACE is a novel tool that is practical, relevant and reliable, and developed in partnership with consumers and health service providers. The tool can be used by health service providers, researchers and consumer agency groups to assess opportunities for consumer engagement offered within the physical environment of cancer services. The ACE tool has the potential to be used as a guide for enhancing consumer engagement opportunities and for research purposes. Further evidence about the validity of the tool is required, including criterion‐related validity and utility in other health settings.

**Patient/Public Contribution:**

This project is part of a wider ‘CanEngage’ project that includes a consumer investigator and is supported by a consumer advisory group. This study was completed in active partnership with members of a consumer advisory group from diverse backgrounds. Feedback was sought from the members throughout the development process of the tool with findings discussed with the CanEngage group members in scheduled meetings.

## 1 INTRODUCTION

Improving consumer engagement in healthcare is recognized as a strategy for enhancing the safety and quality of care.^1^ Consumers are defined as patients and their families or carers who are current, past or potential users of health services.^2^ Consumer engagement is the active involvement of consumers in healthcare decision‐making and can occur across a spectrum from information provision to consultation, involvement and consumer‐led decision‐making at individual, service and system levels.^3^ With a growing desire to more actively engage consumers in healthcare decision‐making and processes, researchers have examined the relationships and interactions that contribute to better engagement between service providers and consumers.^4^, ^5^, ^6^, ^7^, ^8^ Less attention has been paid to the way in which the physical environment in healthcare settings may play a role in the degree and quality of consumer engagement in their care. Contemporary literature has shown the importance of the physical environment in healthcare settings for improving patient health and care outcomes, but a number of findings point to the role of the physical environment in also creating a space for engagement (or barriers to this engagement).^9^, ^10^, ^11^, ^12^, ^13^ For example, research has demonstrated the potential for information about strategies for fall prevention, displayed at the patient's bedside in the hospital, to impact positively on patient and carer engagement in fall prevention planning.^14^ Similarly, patient engagement in their care is also impacted by physical signage including the placement of hand hygiene reminders at the patient's bedside.^15^

The physical environment of health services refers to the layout and content of the location in which health services are provided.^16^ Activities that support engagement can include posters and displays that aim to encourage consumers to interact with the service on a day‐to‐day basis through particular activities or events or through membership of a board or governance committee.^17^, ^18^, ^19^ To discern the extent of success in how a physical environment provides optimal opportunity for consumer engagement, an evaluation is required to capture the availability and extent of opportunities for engagement.^20^, ^21^, ^22^ Audit tools have been developed to assess consumer engagement, but these tools are limited to the assessment of the process of engagement, outcomes of engagement, and health services compliance with the accreditation requirements, such as provision of information to consumers informing them of their healthcare rights.^23^, ^24^, ^25^ Further tools assess patient experience and satisfaction with care^25^, ^26^, ^27^ but they do not assess the physical environment within the health service as a factor that may create opportunities for consumer engagement. To support health service providers and researchers to evaluate the opportunities for consumer engagement in health services environments, we aimed to develop and validate an environmental audit tool designed to assess the extent to which the physical environment in health services creates and supports opportunities for consumer engagement.

## 2 METHOD

**Figure 1:**
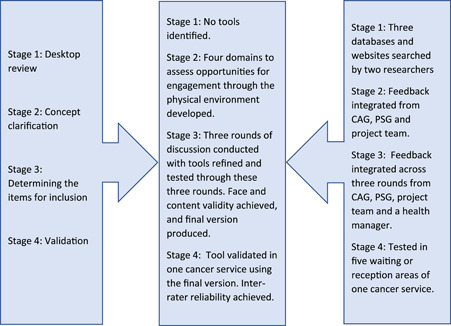
Development and validation process of the Audit for Consumer Engagement tool (consumer advisory group [CAG]; project steering group [PSG])

An iterative, team‐based approach was used to develop and refine the Audit for Consumer Engagement (ACE) tool through four stages grounded in the engagement framework developed by Carman et al.^3^ The consumer engagement framework developed by Carman and colleagues is a widely recognized framework and classifies consumer engagement across a continuum of consumer consultation through to partnership in healthcare decision‐making in direct care, organizational design and policy making.^3^ Hence this framework was used to understand the level of engagement and approaches used to promote engagement with consumers.^17^ Our team consisted of seven health professionals from a range of disciplines (including allied health, pharmacy, nursing, psychology and social work). The team worked in partnership with a consumer advisory group with diverse representation, a wider academic and clinical project team and a project advisory group of health system partners. The consumer advisory group consisted of five members from diverse cultural backgrounds (Australian, Vietnamese, Burmese and middle Eastern) who were cancer service consumers. The project steering group consisted of eight members who were health service managers, policymakers and health professionals from various national and state level health departments/agencies and multicultural healthcare units from public health districts. Ethics approval was granted by a National Health and Medical Research Council (NHMRC) accredited Human Research Ethics Committee (Approval number: 2020/ETH00965). Figure 1 briefly describes the development and validation process of the ACE tool.

### 2.1 Stage 1: Desktop review

A desktop review is a narrative literature review of published and grey literature.^28^ Desktop review method was selected over scoping review or environmental scan as it is an accepted method of conducting a review of published and unpublished work and other materials as the first stage in a tool development process.^29^ A desktop review was conducted by two researchers (A. C.; B. N.) to identify existing tools and indicators that assessed consumer engagement opportunities in the physical environment of health services.^29^ A search was conducted across three search engines (PUBMED, Web of Science and Google Scholar) using keywords and synonyms for ‘consumer engagement’ ‘audit tools’ and ‘physical environment’ in healthcare that were published since 2011 and were in English. Grey literature search was conducted through the websites of major healthcare improvement agencies in Australia, Canada, New Zealand, the United Kingdom and the United States (Australian Commission on Safety and Quality in Health Care [ACSQHC], Canadian Patient Safety Institute, Health Quality and Safety Commission New Zealand, National Health Services [NHS] and Agency for Healthcare Research and Quality [AHRQ]). Potentially relevant articles were exported and tabulated by the two researchers (A. C.; B. N.). The articles were then collated and subject to a further process of review and discussion between the two researchers against the eligibility criteria. To be eligible for inclusion the articles had to report on a tool, scale, item(s) or indicator(s) that assessed or discussed consumer engagement in a health‐related context with reference made to engagement with the physical environment or the impact of the physical environment on the engagement process. The systematic reviews that synthesized these tools/scales/item(s)/or indicator(s) were also included. Any discrepancies between the researchers were resolved through discussion with a third team member (R. H.).

The desktop review did not identify any tools that assessed consumer engagement opportunities in the physical environment but identified tools that assessed consumer engagement and consumer experiences of engagement.^4^, ^6^, ^7^, ^25^, ^29^, ^30^, ^31^, ^32^, ^33^, ^34^, ^35^ The items within these tools (presented in Table 1) that focused on physical or environmental characteristics were reviewed with the following key domains identified: information provision and access; physical access to service; navigation; staff support and attention to the diversity of the population.^25^, ^30^, ^31^, ^32^, ^33^, ^34^, ^35^, ^36^, ^37^ The presence of these domains in the physical environment may enhance the opportunity for consumer engagement.^21^, ^38^ For example, the availability of health information material in waiting rooms in General Practice clinics has been linked with patient involvement in their care.^34^, ^39^, ^40^ Moreover, consideration of diversity is essential for designing health services and this may include signage in different languages or symbols.^32^ Finally, items measuring environmental planning decisions that encouraged consumer engagement were identified from research evaluating consumer engagement frameworks.^17^ Initial discussion within the team of five researchers (A. C.; B. N.; R. H.; R. W.; H. S.) led to adaptations of these items to assess the opportunities for consumer engagement offered by the physical environment of health services through the four domains described in Stage 2.

**Table 1:** Summary of tools with relevant items for physical environment

Authors	Setting	Tool/questionnaire/survey (if applicable)	Aims	Relevant domains/metrices/items assessed
Maskell et al.^34^	General practice clinics waiting rooms	Questionnaire—Patient perception and use of Health Education Materials	To examine patients' perception of and engagement with health education materials displayed in waiting rooms	Health information material
Penry‐Williams et al.^30^	General practice clinics waiting room	Questionnaire and Observations	To examine patient (or accompanying person) and clinician engagement with waiting room health information, including televised health messages	Reading magazine or other health information available, using mobile device, watching a televised health or well‐being program
McDonald et al.^35^	Hospital outpatient rehabilitation waiting room	Purpose‐designed instrument	To assess if the health information is accessed by consumers	Access to health information material
Kline et al.^31^	Mental health setting	Client Engagement and Service Use Scale (CENSUS)	To assess engagement with treatment offered	Access to and availability of staff support
Lachter et al.^25^	Gastroenterology outpatient clinics	Patient questionnaire	To assess experience of patients of the practice area	Staff support, accessibility to reception staff, accessibility of the reception area, accessibility and quality of reading material and health information material
Benton and Overtree^32^	Mental health setting	Items or elements of health service design	To determine physical design elements for multicultural clinics	Diversity consideration, navigation, signage, staff support
Choi and Lee^36^	Aged care setting	Person Directed Care (PDC)	To measure the person‐centred care for residents in nursing home in Korea	Access to physical environment
Sullivan et al.^37^	Community living Centres	Person Directed Care (PDC)	To test and revise the staff assessment of person‐centred care	Access to physical environment
Australian Commission on Safety and Quality in Health Care^33^	Multi purpose health‐services and hospitals	Tool to monitor compliance with NSQHS Standards	To assess compliance with National Safety and Quality Health Services Standards	Healthcare rights, feedback and complaints, attaining to diversity

### 2.2 Stage 2: Clarifying concepts through group consensus

Stage 2 clarified the domains for inclusion in the audit tool. Carman and colleagues' framework,^3^ coupled with the domains identified in the desktop review, collectively formed the basis for the domains included in the ACE tool. These domains were examined by the research team and feedback from members of the consumer advisory group, wider project team and project steering group was sought and integrated. The resulting ACE tool comprised four domains: (1) access and signage information; (2) resources for consumers; (3) resources to support diverse engagement, and (4) consumer engagement events or activities. A description of the domains is provided in Table 2.

**Table 2:** Description of the domains of the Audit for Consumer Engagement Tool

Domain	Description
Access and signage information	Assesses level of access and contact with staff available in the physical environment including information on signage for navigation and directions.
Resources for consumers	Assesses type of resources available for consumers, languages in which these resources are available, and support offered to consumers via these resources.
Resources to support diversity	Assesses whether consideration is given to the diverse consumers with language and other support needs and mechanisms to address these needs.
Consumer engagement events or activities	Assesses the extent to which information is sought from or provided to consumers that may lead to their participation in decision‐making or improvement in health services.

### 2.3 Stage 3: Determining the items for inclusion in the ACE tool

Stage 3 involved an iterative process of refinement and framing of the domains and response options for each of the four domains and items within each domain together with scoring instructions for environmental observers. Initial criteria for domain format were discussed between the five researchers (A. C.; R. H.; B. N.; R. W.; H. S.) with feedback integrated from the project team and consumer advisory group. The final criteria used to determine audit format were as follows: (1) Rating response formats would be appropriate to domain content; (2) The assessment would involve no more than two pages to facilitate ease and speed of completion; (3) The audit tool would provide comprehensive, unambiguous, definitions of all terms and concepts; and (4) A user guide would be available to further support consistency in observation and scoring.^41^

One researcher (A. C.) developed the prototype tool with initial items (*n* = 22) for four domains. The research team of five researchers (A. C.; B. N.; R. H.; R. W.; H. S.) met on four different occasions to iteratively review the prototype tool and to refine the domains and items within each domain before the first audit. Following this, the first audit was conducted by two researchers (A. C.; B. N.) using this first refined version. After completing the first audit, the two observers (A. C.; B. N.) discussed their findings with each other and took notes on the applicability, ambiguity and relevance of the domains and items within each domain. These findings were then discussed with the three other research team members (R. H.; R. W.; H. S.) in three separate meetings to further refine the tool. During this stage, the tool was also reviewed by other members of the research team (C. W.; E. M.), the consumer advisory group, the project steering group and the wider project team. Feedback received through this wider consultation was integrated within the three meetings to further refine the tool and a second refined version was developed. Following this, a second audit was conducted by two researchers (A. C.; B. N.) using the second refined version.

After completing the second audit, findings and notes made by the two reviewers were discussed in three separate meetings with the team of seven researchers, integrating written feedback from the consumer advisory group, wider project team and one health manager external to the team. The final version was then developed and used for validation of the tool. In further developing these items through these discussions, the consumer engagement framework guided decision‐making together with consideration of the context in which these tools would be used, ease of understanding for tool users, and brevity.^29^

The development process was designed in a way to address face and content validity. Face validity is the validity of the tool at face value and this examines if the tool is measuring what it intends to measure.^42^, ^43^ Content validity is the examination of how well the domains of interest have been sampled by the items in the tool.^42^, ^43^ Together, face and content validity examine if the intended subject matter has been clearly addressed and whether the range of aspects is adequately covered.^42^ Determination of face and content validity was based on feedback from five consumers (experts by experience) from diverse backgrounds who were part of the consumer advisory group, eight health service staff representing diverse organizations who formed a project steering group, the research project team consisting of academics and health professionals, and one health service manager external to the project team. The number of experts, including experts by experience, meets the minimum requirement for determining content validity.^44^ Each version of the tool developed was sent to these members for review. For each version of the tool, they were asked to provide qualitative feedback on the domains and items and their relevance to consumer engagement, potential missing content, any ambiguity, and ease of understanding. While content validity was not determined using a quantitative approach, qualitative feedback received demonstrated all experts, including experts by experience (consumers) and healthcare professionals, agreed on the content validity of the final version of the tool.

The qualitative method was used to assess content validity due to the intangible nature of the domains and items used in the tool.^45^ In addition, no previous tools exist to compare the ACE tool for quantitative assessment. Qualitative feedback on the tool was received via email. This qualitative feedback was collated by one author (A. C.) and discussed with four other authors in regular meetings (B. N.; R. H.; R. W.; H. S.) and changes were made as required. This iterative process was followed until the final draft was produced.

### 2.4 Stage 4: Validation of the ACE tool

Validation of the final version of the tool was conducted by two researchers (A. C.; B. N.) independently to enable preliminary inter‐rater reliability assessment between the two reviewers. The final paper‐based tool developed (Figure 2) was also transferred to a web‐based Qualtrics survey platform to facilitate data capture, storage and download. Pilot data were collected using the paper‐based tool as well as the Qualtrics survey tool.

Figure 2Audit for Consumer Engagement Tool

**Figure:**
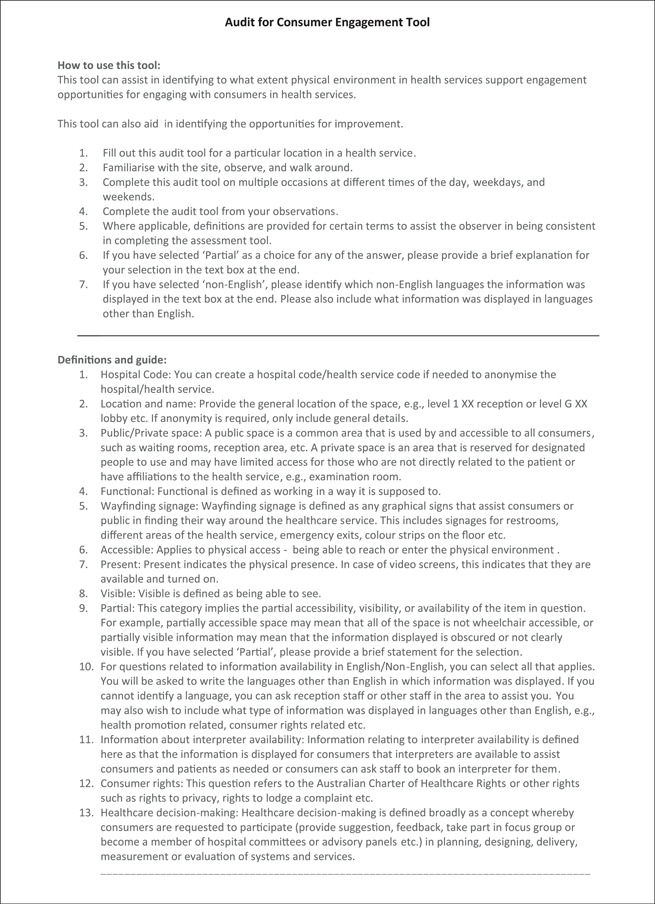

**Figure:**
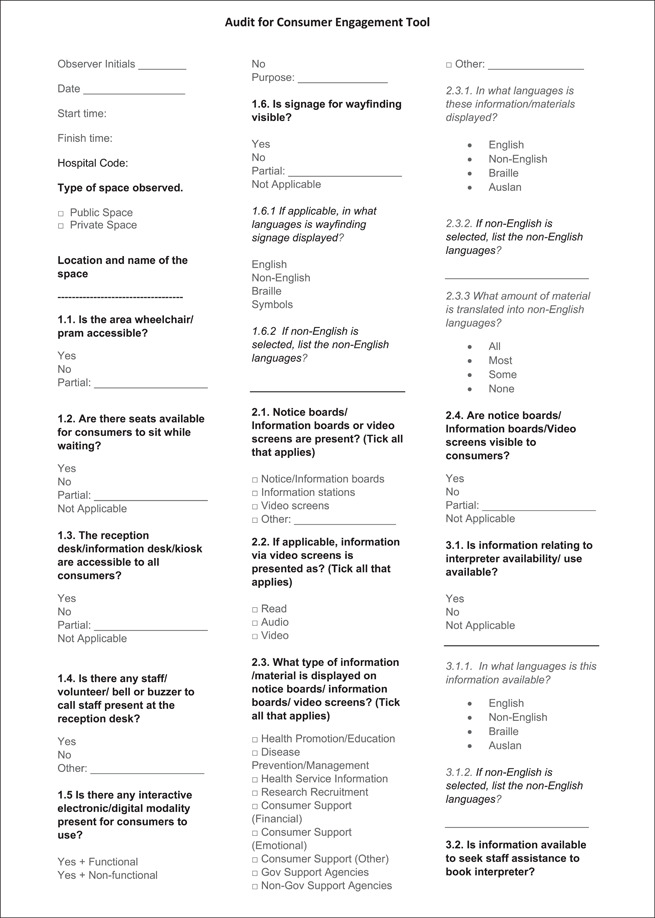

**Figure:**
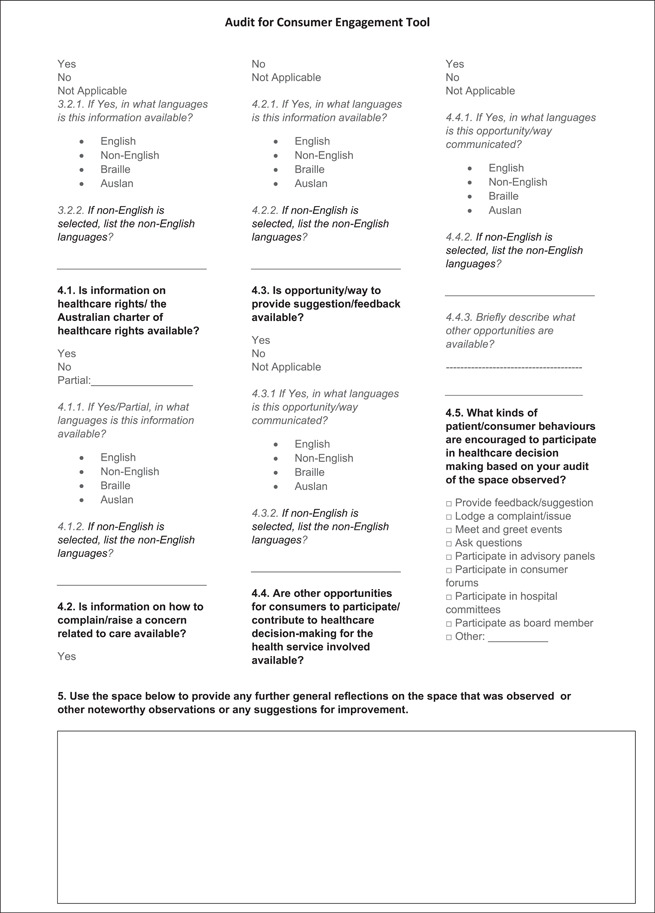

The final tool was applied in five waiting rooms and reception areas of a large cancer service in one public health district in New South Wales (NSW) across both inpatient and outpatient settings. Well over one‐third of the population residing in this health district were born overseas.^46^ This cancer service provides inpatient, outpatient and ambulatory care to patients with cancer and blood disorders delivering surgical, chemotherapy, radiotherapy and palliative care. Using the final version of the tool, two researchers (A. C.; B. N.) collected data in the same environment at the same time, although independently of each other, in order to facilitate analysis of inter‐rater reliability. Due to COVID‐19‐related restrictions in place, inpatient oncology wards were not audited and staff at the participating service were asked to inform the research team of any notable changes to the physical environment.

### 2.5 Inter‐rater reliability analysis

A *κ* score was calculated using the data collected from five separate physical environments using the final version of the ACE tool. Data were analysed in SPSS (IBM Corporation, Version 27) and the *κ* score was calculated for 10 sets of audits conducted to test for inter‐rater reliability (five audits per researcher). The inter‐rater reliability score was calculated for each of the four domains separately.

## 3 RESULTS

### 3.1 The ACE tool

The final ACE tool (Figure 2) consisted of 17 main multiple‐choice questions reflecting the four main categories (access and signage information, resources for consumers, resources to support diversity and consumer engagement activities or events) and one free‐text entry opportunity for recording observations and suggestions for improvement. The questions measured the extent to which the physical environment encouraged consumer engagement with the materials, and provided opportunities to interact with staff and health services. Responses were recorded using relevant single‐choice (yes/no or not applicable) or multiple‐choice options. The items in the audit tool were kept broad for the purpose of their application to diverse physical environments within health services.

### 3.2 Face and content validity

Feedback collected from members of the consumer advisory group, project steering group, project team, and a health service manager indicated that domains and items within each domain were relevant to the aim of the tool. Feedback received through these iterative processes resulted in changes to items (e.g., removing items related to the patient liaison officer; adding an item to assess if the staff were present at the reception desk/kiosk or if any buzzer/bell was present to seek staff), regrouping and clarification of some items (e.g., collecting data separately for feedback and complaints; regrouping type of information displayed into broader categories), adding definitions for terms (availability, functional, physical environment) and limiting the narrative text entry to one section (the first version of the tool had three text box entries). Feedback also highlighted using the text box entry for improvement suggestions. Members of the consumer advisory group identified that items related to the patient liaison officer were not applicable. They also assisted with the framing of questions on interpreter availability and booking and suggested including definitions of key concepts (such as ‘functional’ highlighted above). Consumers also identified the need for assessment of the level of readability of the resources displayed; however, this was not included in the current version as it was beyond the scope of this tool.

### 3.3 Inter‐rater reliability

The final data set consisted of 10 audits (5 audits completed by each researcher) from five waiting rooms and reception areas of one cancer service. The audit time varied with a minimum of 30 min to a maximum of 45 min spent in each area. The *κ* score for each of the four domains of the audit tool is outlined in Table 3. Scores indicated substantial preliminary agreement between the two researchers for all four domains with an average of 86% agreement across the instrument.^47^, ^48^

**Table 3:** *κ* score for four domains of the Audit for Consumer Engagement Tool

Element of Audit for Consumer Engagement Tool	*κ* score
(a) Access and signage information	0.842
(b) Resources for consumers	0.703
(c) Resources to support diversity	1.00
(d) Consumer engagement events or activities	0.890

## 4 DISCUSSION

The ACE tool is a novel tool that can be used to assess the physical environment for opportunities for and barriers to consumer engagement. The ACE tool is a simple, observational checklist that can be utilized by staff in health services, consumer advocacy agencies and researchers to establish whether an environment actively supports consumer engagement. Although the role of the physical environment in impacting healthcare delivery and outcomes is recognized,^11^ the ACE Tool addresses a gap in instruments available to assess how well the physical environment encourages consumer engagement and the opportunities for improvement. Our analysis indicates that the tool has face and content validity and good preliminary inter‐rater reliability across its domains when applied in cancer services and now requires further validation in a range of specialities and services. The next step should involve collection of criterion‐related validity data including the extent to which scores on the tool discriminate those where consumers are currently engaged in health service planning from those where engagement is less apparent.

The process of developing the ACE tool revealed the need to include items to assess the availability and provision of information in various languages and for those with diverse communication needs to ascertain opportunities for engagement for a diversity of populations. Consideration of diversity is increasingly recognized as critical to improving the equity of the quality of healthcare, nationally and internationally.^49^, ^50^, ^51^ Inequities in consumer engagement opportunities and experiences amongst people from diverse backgrounds (e.g., ethnically diverse people, people with disabilities and Indigenous populations) are identified as contributing to variable healthcare quality and experiences.^50^, ^52^ An environment that is sensitive and responsive to the needs of diverse health consumers is a necessary step and likely precondition to addressing these inequities in healthcare engagement and, ultimately, quality. Such an environment would likely include; resources developed in collaboration with relevant culturally and linguistically diverse (CALD) communities who frequent the service, and available in a variety of languages, culturally responsive and sensitive text (sensitive to use of certain colours and fonts in some languages), using approaches such as minimal language approaches and ensuring that consideration is given to the variability in levels of health literacy between service users.^53^, ^54^ The ACE tool also captures the diverse ways in which information can be displayed in the waiting areas. This is important due to the increasing use of digital technology and the digital transformation of health services.^55^

### 4.1 Implications

The ACE Tool has applications in practice and research. The use of this tool during health facility design may enable service designers to proactively address those aspects of the physical environment that optimize opportunities for consumer engagement. The Australasian Health Facility Guidelines provide guidelines for the design of a health facility that supports implementation of contemporary models of care such as patient‐centred care and consumer participation in healthcare decision‐making.^56^ The guidelines recommend the use of tools to assess areas of facility design such as Wayfinding whereby guidance on how consumers can be helped to navigate a health service is provided.^56^ Use of the ACE tool can assist with implementation of guidelines and further identify potential barriers to engagement.

With increasing focus on consumer engagement in healthcare decision‐making and requirements for health providers to demonstrate the steps taken towards this,^57^ the ACE tool may be used to demonstrate organizational commitment to enhancing consumer engagement. By collaborating with consumer representatives and staff to complete the assessment, service providers can demonstrate their commitment to improving consumer engagement and compliance with regulatory standards and identify specific actions to deliver outcomes that are meaningful to consumers. Commitment in the form of resources and staff time is needed. This commitment, in turn, requires leadership buy‐in to ensure that the tool progresses beyond the identification of opportunities to the realization of change for improvement in consumer engagement.^58^

The ACE Tool may be applicable to a range of settings beyond cancer services and requires further analysis to explore the relevance and validity of the tool when applied in different settings. Further work may seek to explore whether the expansion of the tool to consider and quantify levels of engagement (from information provision and consultation through to participation and consumer‐led decision making), and to provide analysis regarding the accessibility and/or cultural appropriateness of the information provided, including considerations around health literacy in material provided or displayed in the physical environment, would also be valuable.

### 4.2 Strengths and limitations

Carman and colleagues’ engagement framework provided a theoretical basis for the development of the ACE tool.^4^, ^17^ This is a widely used and recognized engagement framework that describes the involvement of consumers at the individual care level as well as their participation in higher‐level health system and service level decision‐making. It aligns with other engagement frameworks including the IAP2 (International Association of Patient Participation) Spectrum of Public Participation, Arnstein's Ladder of Citizen Participation, the National Safety and Quality Health Services (NSQHS) Standard 2: Partnering with consumers, the National Institute of Health and Care Excellence (NICE) framework for community engagement, and those used by many health services to guide their consumer engagement policies.^18^, ^57^, ^59^, ^60^, ^61^ Despite its strengths, the use of this framework as a basis for the tool may have also shaped the tool's focus and content.

In developing the ACE, repeated discussions and consultations between the research team, consumer advisory group, project team and project steering group addressed the critical issues of clarity and definitions for the terminologies used. By providing definitions and guide at the outset and using broad domains, the tool could be easily understood and interpreted with success demonstrated via a high preliminary inter‐rater reliability score.^29^ Further, the involvement of the consumer advisory group, project steering group and a health service manager provided the opportunity to integrate feedback from service users as well as service planners. This process also made the audit tool more robust and applicable to diverse health services contexts. Nonetheless, it is important that further environmental data are collected by observers who are independent of the tool development process to firmly establish reliability.

A further limitation of the study is the exclusive focus on face and content validity, which are weaker forms of psychometric validation. Nonetheless, face and content validity are critical to acceptability and feasibility particularly in clinical settings (e.g., cancer setting) with research suggesting that this form of validity is a hurdle requirement before other forms of validation data are collected.^43^ A desktop review was conducted instead of a scoping review and this may have shaped our findings relating to the identification of relevant tools in stage one. Broad categories were used to classify and categorize the information collected, and this could likely miss more nuanced differences between environments. Moreover, due to COVID‐19‐related restrictions, the tool was only validated in public waiting and reception areas in one cancer service. In the future, the utility of the tool for use in inpatient wards and in private consulting rooms should also be tested. While the tool has implications for use by consumer representatives, the tool was not tested by the consumers. The tool may also have limited applicability for some specialized health services and populations such as mental health services and for those with cognitive impairment.

## 5 CONCLUSION

With increasing focus on consumer engagement in the process of enhancing the quality and safety of healthcare delivery, it is essential to recognize the role of the physical environment within health services as a facilitator or barrier to consumer engagement. This paper described the development and initial content validation of a novel tool that can be used by various stakeholders to identify and assess opportunities for consumer engagement that are supported by the physical environment of health services. Collaboration with consumers and health services underpinned the development process and resulted in a practical, relevant and reliable tool. This tool has further potential to be used as a guide for enhancing consumer engagement opportunities and for research purposes. This tool had demonstrated content validity at one cancer service and may now be applied to other health services to demonstrate this form of validity across a diverse range of health settings. Additionally, criterion‐related validity data should also be collected to determine whether environments scoring more highly on the ACE report have higher levels of consumer engagement.

## AUTHOR CONTRIBUTIONS

Ashfaq Chauhan and Reema Harrison conceptualized the study. Ashfaq Chauhan and Bronwyn Newman completed the data collection. Ashfaq Chauhan completed the data analysis and draft of the manuscript. All authors discussed the draft and provided feedback. All authors approved the final version for submission.

## CONFLICT OF INTEREST

The authors declare no conflict of interest.

## ETHICS STATEMENT

Ethics approval was granted by a National Health and Medical Research Council (NHMRC) accredited Western Sydney Local Health District Human Research Ethics Committee (Approval number: 2020/ETH00965).

## Data Availability

The data that support the findings of this study are available on request from the corresponding author. The data are not publicly available due to privacy or ethical restrictions.
